# Is Blood a Good Indicator for Detecting Antimicrobials in Meat? Evidence for the Development of In Vivo Surveillance Methods

**DOI:** 10.3390/antibiotics9040175

**Published:** 2020-04-12

**Authors:** María Jesús Serrano, Olga Mitjana, Cristina Bonastre, Alicia Laborda, María Victoria Falceto, Diego García-Gonzalo, Eunate Abilleira, Janire Elorduy, Alain Bousquet-Melou, Luis Mata, Santiago Condón, Rafael Pagán

**Affiliations:** 1 Instituto Agroalimentario de Aragón-IA2 (Universidad de Zaragoza-CITA), 50013 Zaragoza, Spain; mjserran@unizar.es (M.J.S.); omitjana@unizar.es (O.M.); cbonastr@unizar.es (C.B.); alaborda@unizar.es (A.L.); vfalceto@unizar.es (M.V.F.); Diego.Garcia@unizar.es (D.G.-G.); scondon@unizar.es (S.C.); 2 Public Health Laboratory, Office of Public Health and Addictions, Ministry of Health of the Basque Government, 20013 Guipuzkoa, Spain; e-abilleira@euskadi.eus (E.A.); j-elorduy@euskadi.eus (J.E.); 3 INTHERES, Université de Toulouse, INRA, ENVT, 31300 Toulouse, France; a.bousquet-melou@envt.fr; 4 Department of R&D, ZEULAB S.L., 50197 Zaragoza, Spain; lmata@zeulab.com

**Keywords:** antibiotic, sulfonamide, quinolone, HPLC-FLD, LC–MS/MS, meat, blood

## Abstract

The introduction of antimicrobial residues in the food chain has a significant impact on human health. An innovative solution to avoid their presence in meat is the adaptation of current control methods for use with in vivo matrixes. Thus, the aim was to obtain paired blood and muscle samples from pigs treated with some of the main antimicrobials currently used in veterinary medicine (oxytetracycline, sulfamethoxypyridazine, enrofloxacin, amoxicillin), and to compare their rate of depletion in both matrixes. Antimicrobial concentrations in paired samples of blood and muscle were determined by liquid chromatography with tandem mass spectrometry (LC–MS/MS) or high performance liquid chromatography with fluorescence detection (HPLC-FLD). A comparison between values obtained in muscle and blood showed a similar distribution in both matrixes for oxytetracycline; for sulfamethoxypyridazine, a similar decrease rate but a concentration three times higher in blood compared to muscle was found; for enrofloxacin, we found significant differences in the rate of depletion, with similar antimicrobial concentrations in both matrixes with values close to the maximum residue limit (MRL) and higher amounts in muscle for values that lay considerably over the MRL. Conversely, amoxicillin depletion was so rapid that its appearance in carcasses does not seem to pose a risk. Therefore, blood would be a feasible matrix for the development of new in vivo tests.

## 1. Introduction

The World Health Organization regards antimicrobial resistance (AMR) as one of the major threats to health, in which humanity will have to face in the next decades, as it involves a significant decrease in antimicrobial effectiveness. If worst comes to worst, we could be heading toward a post-antibiotic era, in which common illnesses could easily lead to great economical and human losses. O’Neill [1] reports the severity of this issue—by the year 2050, if nothing is done in the next 30 years, an accumulated loss of EUR 88 billion is predicted, along with 10 million annual deceases (a mortality rate greatly exceeding that of cancer).

Although AMR is a problem of major concern in human medicine, its origin is not restricted to that area of activity. Veterinary medicine is actually the main consumer of antibiotics. According to the second joint report on the consumption of antimicrobial agents in humans and food-producing animals, nearly 70% of all the antibiotic consumption in the EU in 2014 was in the area of animal production [2].

In terms of meat consumption and meat safety, AMR microorganisms of pure animal origin are not the only source of concern—traces of antimicrobials administered by humans to animals can likewise still be present in meat and its derivates. This problem has been addressed in the EU by the establishment of maximum residue limits (MRL), defined by Regulation (European Economic Community (EEC)) No. 2377/90 [3]. Moreover, the requirements established by Article 12.3 of Directive 2001/82/EC [4] point to the need of including an indication of the withdrawal period, whereas the EMA (European Medicines Agency) has published a guideline to establish withdrawal periods for edible tissues of food-producing animals in the EU [5]. These policies enable both the meat sector and health authorities to develop exhaustive control procedures for meat products.

A wide range of methods for the detection of antibiotic residues in meat are currently available—biological screening methods were the earliest forms to have been developed [6], and their use is still widespread, as they are cost-effective and have a broad spectrum [7]. As these are qualitative methods, when a positive sample is found it is necessary to carry out confirmatory methods on the basis of the physicochemical properties of antibiotic molecules (High performance liquid chromatography (HPLC), Liquid chromatography with tandem mass spectrometry (LC–MS/MS) [8].

Although both the screening and confirmatory methods indeed fulfill the surveillance requirements posited in Council Directive 96/23/EC [9], these are post-mortem methods that require a muscle or meat sample from slaughtered animals. The detection of positive samples entails considerable financial losses resulting from the confiscation of carcasses, along with severe fines. Although economic consequences are of great significance for the meat sector, a strong environmental impact is likewise associated with the production of pigs that end up as waste, not only in view of the input such production requires (water, energy, etc.) but also because of its inevitable output (gas emissions, waste water, manure/slurry, and the complicated management of unnecessarily confiscated carcasses) [10]. These, however, might not be the most serious implications deriving from carcasses contaminated with antibiotics. A failure to detect contaminated meat would allow for the incorporation of antibiotic residues into the food chain, thereby leading to consumer intake of those residues, with considerable direct and indirect impact on human health.

An innovative solution to these concerns could be found in the adaptation of current control methods in order to use them on samples from living animals instead. This new approach would require that there be a suitable relationship between the concentration of antimicrobial substance in muscle and matrixes easily obtained from living animals.

Several studies on antibiotic concentration in muscle [11] and on other matrixes such as blood plasma [12,13] have been published, along with articles about the relation between antimicrobial residues in muscle and other matrixes that could be obtained in vivo: hair [14], urine [15], and feathers [16]. Such further exploration involves detailed studies of the pharmacokinetics and the relation between the concentration of the target substances in muscle as well as in other tissues or fluids easily obtained from living animals. Such correlations would be dependent on the pharmacokinetics associated with each antimicrobial family.

The aim of this study was to create an experimental sample bank containing naturally tainted paired blood and muscle samples from animals that had already been injected in vivo with varying antimicrobial concentrations from several antimicrobial families—different pharmacokinetics (quinolones, sulfonamides, β-lactams, and tetracyclines) would correspond with different days within the withdrawal period. This working procedure would allow for a direct comparison between the concentrations of antimicrobial residues in both matrixes, and would thereby help us ascertain whether blood is an accurate indicator of the presence of antibiotics in muscle or not, and whether it represents a good option for the development of in vivo surveillance methods.

## 2. Results and Discussion

The development of new in vivo antimicrobial detection methods (or the adaptation of current post-mortem ones to ante-mortem techniques) in meat requires further knowledge. This deepening involves detailed studies about the pharmacokinetics and the relationship between the concentration of the target substances in muscle as well as in other tissues or fluids easily obtained from living animals. Thus, as a first step, we gathered a collection of paired muscle and blood samples from animals that had been injected in vivo by certain relevant antimicrobial compounds currently used in livestock.

The antimicrobials chosen for this study were oxytetracycline, sulfamethoxypyridazine, enrofloxacin, and amoxicillin, as they are among the most commonly used antimicrobial substances in animal production in the EU; furthermore, they follow substantially divergent metabolic pathways [17].

In order to obtain the paired samples, piglets were administered with the selected products and slaughtered on different days within the withdrawal period indicated by the manufacturer. After slaughter, samples of blood and different muscle groups were obtained (Table S1).

**Figure 1:**
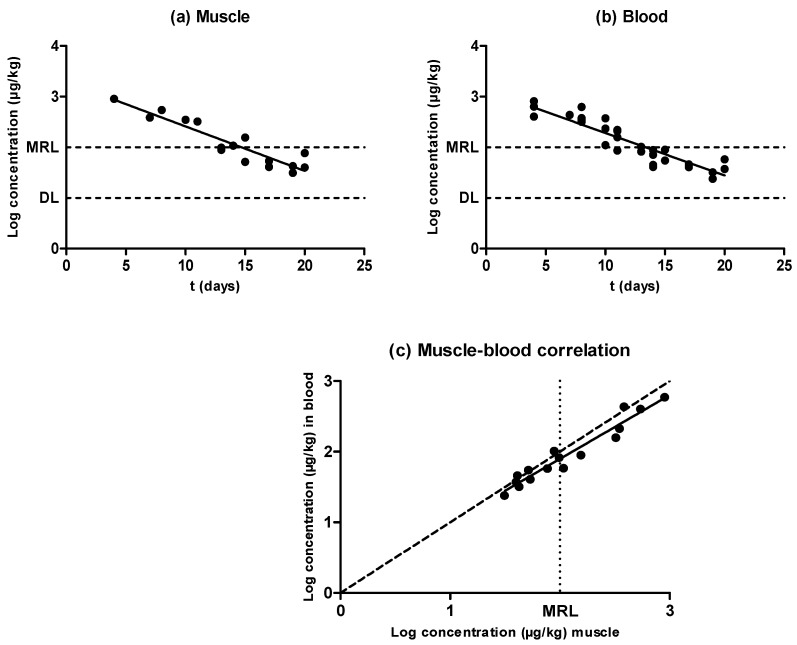
Evolution of the concentration of oxytetracycline in muscle (**a**) and blood (**b**) samples obtained from pigs treated with oxytetracycline and slaughtered at preset intervals within the withdrawal period, determined by liquid chromatography with tandem mass spectrometry (LC–MS/MS). The DL dotted line represents the detection limit of the analytical technique for oxytetracycline, and the maximum residue limit (MRL) dotted line represents the maximum residue limit of oxytetracycline in muscle, as established by Regulation (European Economic Community (EEC)) No. 2377/90. Relationship among the concentrations of oxytetracycline detected in blood and muscle samples (**c**). The bisecting dotted line represents the 1:1 correlation if both matrixes contained the same concentration of oxytetracycline.

**Figure 2:**
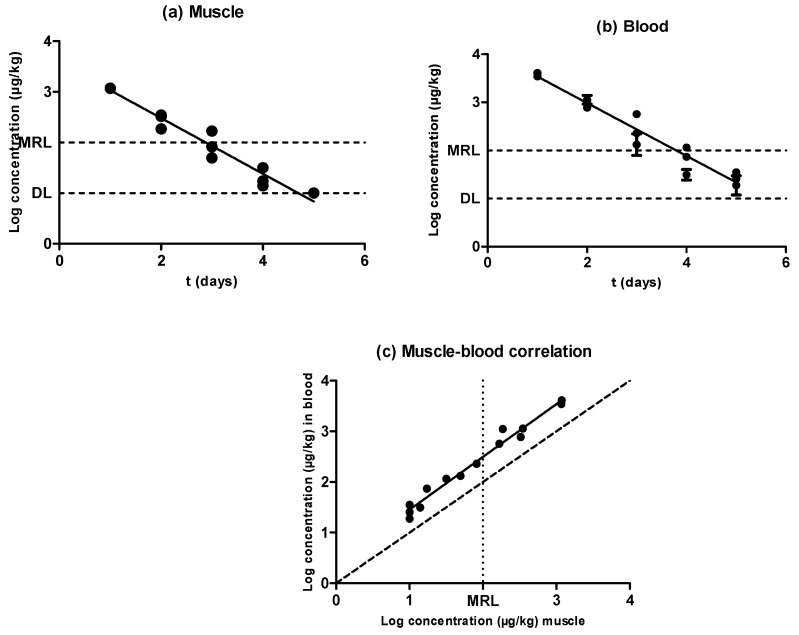
Evolution of the concentration of sulfamethoxypyridazine in muscle (**a**) and blood (**b**) samples obtained from pigs treated with sulfamethoxypyridazine and slaughtered at preset intervals within the withdrawal period, determined by LC–MS/MS. The DL dotted line represents the detection limit of the analytical technique for sulfamethoxypyrydazine, and the MRL dotted line represents the maximum residue limit of sulfamethoxypyrydazine in muscle as established by Regulation (EEC) No. 2377/90. Relationship among the concentrations of sulfamethoxypyridazine detected in blood and muscle samples (**c**). The bisecting dotted line represents the 1:1 correlation if both matrixes contained the same concentration of sulfamethoxypyrydazine.

**Figure 3:**
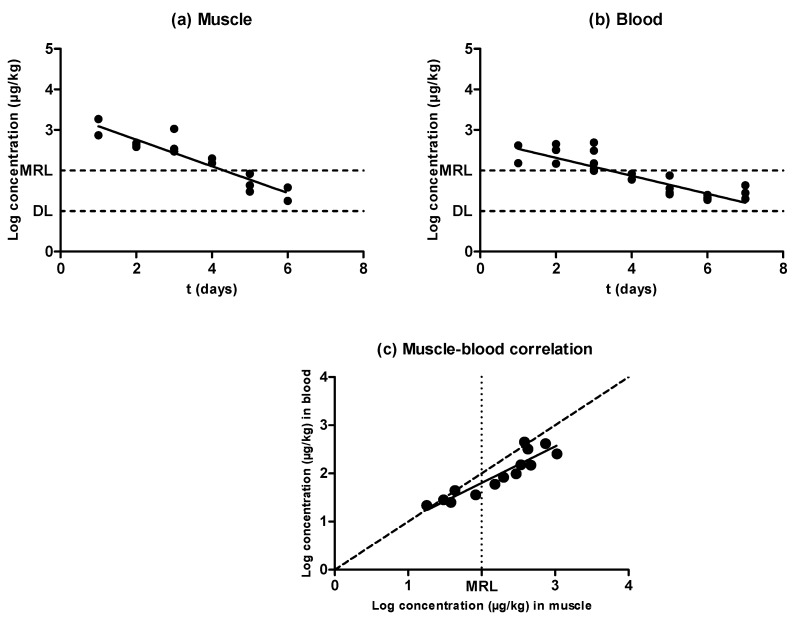
Evolution of the concentration of enrofloxacin in muscle (**a**) and blood (**b**) samples obtained from pigs treated with enrofloxacin and slaughtered at preset intervals within the withdrawal period, determined by LC–MS/MS. The DL dotted line represents the detection limit of the analytical technique for enrofloxacin, and the MRL dotted line represents the maximum residue limit of enrofloxacin in muscle as established by Regulation (EEC) No. 2377/90. Relationship among the concentrations of enrofloxacin detected in blood and muscle samples (**c**). The bisecting dotted line represents the 1:1 correlation if both matrixes contained the same concentration of enrofloxacin.

**Figure 4:**
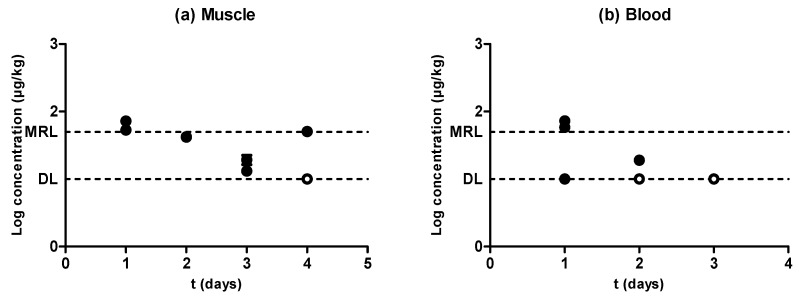

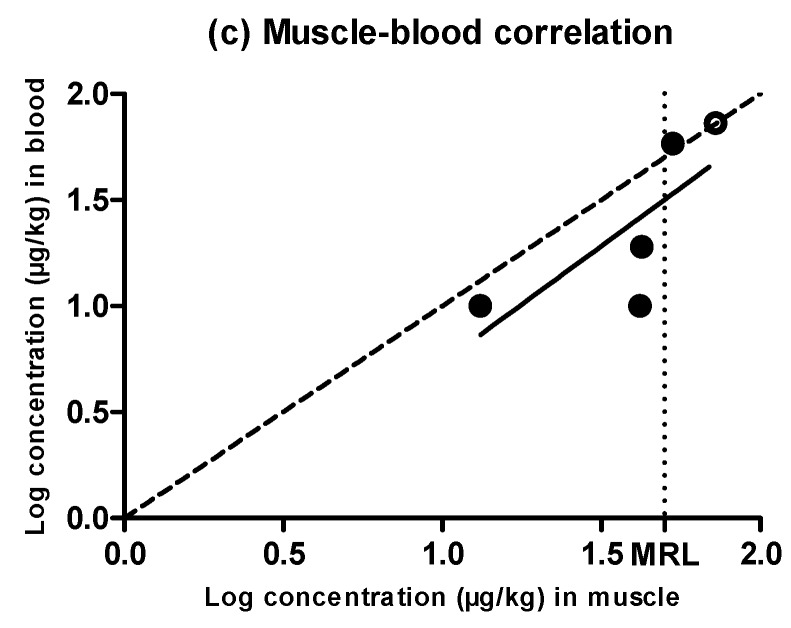
Evolution of the concentration of amoxicillin in muscle (**a**) and blood (**b**) samples obtained from pigs treated with amoxicillin and slaughtered at preset intervals within the withdrawal period. Muscle sample concentrations were determined by high performance liquid chromatography with fluorescence detection (HPLC-FLD), and blood sample concentrations by LC–MS/MS. The DL dotted line represents the detection limit of the analytical technique for amoxicillin, and the MRL dotted line represents the maximum residue limit of amoxicillin in muscle as established by Regulation (EEC) No. 2377/90. Relationship among the concentrations of amoxicillin detected in blood and muscle samples (**c**). The bisecting dotted line represents the 1:1 correlation if both matrixes contained the same concentration of amoxicillin.

To test whether the muscle distribution of the active compound (or metabolites) in each animal was homogeneous, muscles from loins, forequarters, and hindquarters from the 78 treated pigs were collected, and the concentration of each antibiotic was analyzed. Data obtained from these assays (Table S1) showed that in most cases there were no significant differences (*P* ˃ 0.05) among the concentration of antimicrobials within those three locations. Significant differences (*P <* 0.05) were only found when the highest concentration values were obtained; moreover, they were the ones most distant from the MRLs specified for each antimicrobial compound. These slight variations between the amounts of antimicrobial substances retained in different high-level groups could be explained not only by the antimicrobial compounds’ properties and their distribution processes, but also by muscle features such as fat content [18]. In this regard, several studies have been carried out to ascertain whether antimicrobial distribution in muscles is homogeneous or not. Reyes-Herrera et al. [11] found that enrofloxacin levels after oral treatment were higher in breast compared to thigh in poultry, although similar among different breast sections [19]. Nevertheless, due to the slight differences observed in our study, and due to the higher concentrations observed in loins, especially for amoxicillin (Table S1), we only quantified loins in the remaining antibiotic-treated piglets. All the obtained samples were analyzed by HPLC-FLD or LC–MS/MS, thereby ascertaining the evolution of the antimicrobial compound concentration in muscle and blood throughout the withdrawal period (Figure 1, Figure 2, Figure 3 and Figure 4). Because legislation explicitly regulates the MRLs in muscle for all the antimicrobial compounds explored, we included these limits in all of the figures. In any case, these concentrations began at high levels during the first days of the withdrawal period, and reached values lying below the MRLs before the withdrawal period ended (Table S2). Our sample collection was made up of more than 11,000 muscle samples from untreated and treated animals. Over 1500 muscle samples were obtained from untreated animals and were used as a reference for all the analyses performed, and almost 50% of the muscle samples from treated animals displayed levels of a wide range of antimicrobial concentrations lying above the MRLs (Table S2).

### 2.1. Oxytetracycline

Oxytetracycline is usually administered intramuscularly; it has long-lasting formulations, and its distribution is a function of lipid solubility. It is not metabolized to a significant extent in the body; 60% of the dose is eliminated in urine via glomerular filtration, with the other 40% eliminated in the feces [20]. Some studies have revealed an extended mean residence time [13], which, in turn, is reflected in an extended withdrawal period (Table S3).

As shown by Figure 1, antimicrobial concentration decreased as the withdrawal period progressed in both matrixes (muscle samples (Figure 1a) and blood samples (Figure 1b)), and oxytetracycline depletion followed an exponential rate in muscle as well as in blood. When comparing the half-lives of elimination (Table 1), no significant differences were observed between the half-life of elimination in muscle (3.43 days) and that in blood (3.59 days), according to the rates of removal (*P* ˃ 0.05); thus, the pharmacokinetics of oxytetracycline were similar in both matrixes. Consequently, estimations of oxytetracycline concentrations in meat via new detection techniques based on blood samples would not require an adaptation of test sensitivity.

An exponential rate of removal has already been described for oxytetracycline after intravenous and intramuscular administration in sheep [21]. In pharmacokinetics, it is a general rule that the elimination of xenobiotics from living animals (including humans) follows an exponential decay curve [22].

Results showed no differences between blood and muscle oxytetracycline concentrations (Figure 1c). Cars and Ryan [23] found higher concentrations of doxycycline in muscle compared to blood, but similar ones between muscle and blood; Castellari et al. [14] found lower amounts of oxytetracycline in pig and calf muscle compared with hair. These differences regarding tetracycline distribution could be associated with differences in binding to plasma proteins compared to tissular proteins, or with the lipophilic behavior of drugs from the tetracycline group such as doxycycline [23].

### 2.2. Sulfamethoxypyridazine

Sulfamethoxypyridazine is widely distributed throughout the body, particularly in many soft tissues. After attaining a therapeutic concentration in the bloodstream, it is mainly excreted by the kidneys, but also by certain other fluids, such as tears, feces, bile, milk, and sweat, thereby undergoing extensive tubular reabsorption in addition to some enterohepatic recycling [24]. It is catalogued as a long-acting sulfonamide, with an extended half-life in the body, a reason for which it has long withdrawal periods in food-producing animals (Table S3).

As shown in Figure 2, the concentration of sulfamethoxypyridazine in muscle and blood also followed an exponential rate of elimination in both matrixes, with significant differences (*P* < 0.05) appearing in the concentration of this antimicrobial substance among animals subjected to the same treatment and withdrawal period, with no repercussions on the correlation between muscle and blood concentrations (Figure 2c). At any rate, no significant differences (*P* ˃ 0.05) were observed between the half-lives of elimination described in muscle and blood (Table 1), which amounted to 0.55 days. Nevertheless, the connection line drawn over the correlation among muscle and blood concentrations (Figure 2c) was shifted upward from the bisecting dotted line. The concentration was thus always higher in blood as compared to muscle—specifically, around three times higher. Consequently, the design of an alternative in vivo test based on sulfonamide detection in blood should have its sensitivity decreased, as the value obtained from the MRL intercept in muscle and the blood/muscle correlation was approximately 300 ppm. Nevertheless, because the purpose of a screening method is to detect any positive sample with a false-negative rate that should be as low as possible, a slight excess of sensitivity should not be a major issue. A confirmatory method could be additionally applied.

### 2.3. Enrofloxacin

Enrofloxacin absorption is virtually complete when administered intramuscularly because it is metabolized to ciprofloxacin; this is why this compound was also determined when HPLC analyses were carried out. It is primarily excreted via the kidneys, with urine concentrations several times higher than in blood plasma, and small amounts are recovered in feces [25].

Similarly to the previously tested antimicrobial substances, enrofloxacin was eliminated from blood and muscle following an exponential rate (Figure 3a,b). Nevertheless, the concentration of this quinolone was higher in muscle than in blood (*P* < 0.05), and there were significant differences between the half-lives of elimination described in muscle (0.92 days) and in blood (1.90 days) (Table 1). Moreover, a comparison between the antimicrobial levels in blood and muscle is shown in Figure 3c. Although a correlation between the concentration in both matrixes was found, its slope deviated from the dotted line slope, presumably due to rapid antibiotic depletion—enrofloxacin levels reached values close to the detection limit (DL) 7 days after inoculation for muscle and 8 days after inoculation for blood, whereas oxytetracycline levels decreased to the DL 26 days after inoculation in muscle and 25 days after inoculation in blood. This deviation was more pronounced at higher concentrations far removed from the MRL, where higher values for muscle concentration were observed when compared to blood. Cars [26] also described higher quinolone concentrations in muscle than in blood by using a rabbit model. This situation would require an increase in the sensitivity of the detection techniques in order to obtain comparable results in blood and muscle. More precisely, new tests for blood should detect around 60 ppm of enrofloxacin, which corresponds to the value where the MRL (100 ppm in muscle) dotted line intercepts the regression line drawn over the correlation featured in Figure 3c.

### 2.4. Amoxicillin

Amoxicillin is rapidly absorbed when injected in aqueous suspension by the intramuscular route, and after its absorption it is rapidly excreted by urine [27] and by certain other fluids, such as milk [28].

The comparison between the concentrations of amoxicillin in the three muscular groups we studied showed a concentration (*P* < 0.05) of this antibiotic over seven times higher in loins compared with forequarters and hindquarters (Table S1). Although remarkable differences between muscular clusters could be observed, amoxicillin removal from tissues was so rapid that this panorama could profoundly change within short periods of time during the initial portions of the withdrawal period, thereby explaining these discrepancies. Rapid depletion from tissues has also been described in other species following several different methods of administration, for instance, in fish [29] and in sheep [30].

Figure 4 shows a rapid withdrawal of amoxicillin from muscle (Figure 4a) and blood (Figure 4b) that reached amounts close to the MRL (50 µg/kg) in the first day of the withdrawal period. Moreover, muscle and blood samples obtained from animals subjected to the same treatment showed significant differences (*P* < 0.05) between the amounts of retained amoxicillin, although these differences (which were even five times higher) could again be due to the rapid antibiotic depletion from tissues. Although an exponential rate of elimination was observed in muscle (Figure 4a), these differences between replicates, together with a rapid antibiotic depletion, led to a less reliable correlation. This scenario precludes the determination of a solid blood removal rate (Figure 4b). Although the phenomenon of rapid tissue depletion prevented the identification of a clear exponential removal, Hernández et al. [12] have previously described exponential rates of elimination of amoxicillin from piglet blood plasma.

When comparing the concentration of amoxicillin in muscle and blood (Figure 4c), only the highest concentration values lay above the dotted line, whereas the rest of them were considerably below the MRL. This means that, within the first stages of the withdrawal period when antibiotic concentrations were higher, amounts of amoxicillin in muscle and blood were analogous, but the removal of the last traces of amoxicillin followed a slower rate in muscle as compared to blood. Vaden and Riviere [27] also proposed that tissue concentrations of aminopenicillins might be higher than blood concentrations, whereas Cars [26] described higher concentrations in blood compared to muscle, as β-lactam are non-lipophilic drugs and have a weak ability to penetrate cells. These conflicting results might be due to the rapid depletion rates we identified.

### 2.5. Evidences for the Development of In Vivo Surveillance Methods

**Figure 5:**
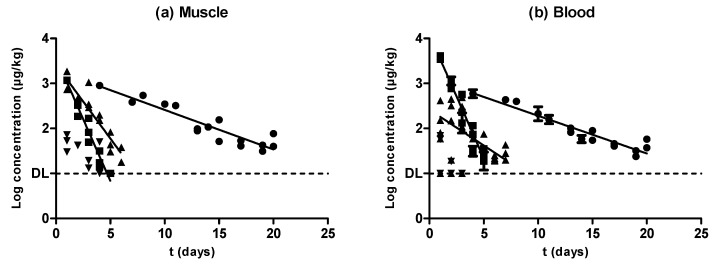
Comparison between the rate of elimination described for oxytetracycline (●), sulfamethoxypyridazine (■), enrofloxacin (▲), and amoxicillin (▼) in muscle (**a**) and blood (**b**) samples.

As described above, almost every antimicrobial substance studied herein followed an exponential rate of removal both in muscle (Figure 5a) and blood (Figure 5b). Oxytetracycline and sulfamethoxypyidazine followed the same elimination rate in muscle and blood, whereas enrofloxacin displayed a higher rate of depletion in muscle compared with blood. Nevertheless, although amoxicillin concentrations decreased as the withdrawal period progressed, no significant correlations were found between those concentrations and the withdrawal period time. The comparison among the different speeds of antimicrobial depletion (Figure 5, Table 1) showed that sulfamethoxypyridazine had the fastest clearance, followed by enrofloxacin and oxytetracycline, both in muscle and blood samples (Table 1). Previous researchers also found slower rates of removal for oxytetracycline compared with other antimicrobial compounds such as sulfadiazine, trimethoprim, flumequine, and oxolinic acid [31].

A wide range of methods for the detection of antibiotic residues in meat currently exist. In terms of common surveillance, screening methods are selected in a first stage. These methods can be divided into conventional and innovative methods [8]. Among innovative methods, the introduction of biosensors as analytical tools in the food and drink industry is a greatly promising development [32]. Nonetheless, conventional methods are currently commercialized for meat, some of the most relevant of which are based on microbial growth inhibition [33].

The design of novel in vivo antimicrobial detection tests based on blood samples would not require any adaptation for oxytetracycline, as the concentrations described in blood were analogous to those found in muscle. Nevertheless, although the correlation between blood and muscle did not point towards the need of modifying the newly developed tests, the study of the effect of blood on the growth of microorganisms will be required, and, therefore, certain modifications in the formulation of the growth media or even previous preparatory steps may be necessary for the adaptation of biological tests to blood.

On the other hand, the amounts of sulfamethoxypyridazine identified in blood were three times higher than those detected in muscle, regardless of concentration; the new test’s sensitivity should thus be decreased in order to lower its detection limit to accurate levels. Several options to obtain this decrease in sensitivity exist, but the option ultimately selected would depend on the type of method used to detect the presence of sulfamethoxypyrydazine. For instance, sulfonamide treatments usually include trimethoprim in their formulation, as this compound increases microbial sensitivity [34]. Likewise, antibiotic detection tests based on biological criteria might include trimethoprim in their formulation in order to increase microbial sensitivity to sulfonamides; thus, a reduction of the trimethoprim concentration added to the growth media formulation would suffice to adapt meat test results to blood test results.

The scenario with enrofloxacin is the opposite. Values around and above the MRL are higher in muscle than in blood; thus, new tests should have their detection limit decreased in order to increase sensitivity. As enrofloxacin is an antimicrobial that inhibits gyrase enzymes participating in the replication of DNA, a good solution to adapt a meat-screening antibiotic detection test based on microbial growth inhibition would be achieved through the reduction of bacterial concentration. Nonetheless, most microbial screening tests use *Geobacillus stearothermophilus* as a target strain. It has already been described, however, that biological tests based on this microorganism’s growth are not able to detect antimicrobial compounds from the quinolone family at levels usually found in food, as it has a low sensitivity to these compounds [35,36]. To overcome this limitation, an alternative microbial screening test based on the inhibition of *Escherichia coli* has also been described with a suitable performance for muscle analysis [33]. In addition, several alternatives to microbial quinolone detection methods in food currently exist. For instance, rapid immunological techniques [37] or instrumental techniques [38] are well described in the literature, offering a certain advantage over microbial screening methods because they identify the concentration of quinolone residues in food. Hence, these kinds of tests would not require any adaptations in blood, as they deliver a concentration value easily correlated with values obtained in muscle (Figure 3c). However, screening tests remain necessary in order to obtain rapid and in situ results that could hardly be achieved with instrumental techniques.

Furthermore, although data obtained from paired amoxicillin samples did not allow for a correlation between muscle and blood concentrations, this antimicrobial withdraws so rapidly from both tissues that residues in meat are unlikely to pose any problem. Amoxicillin describes such a fast depletion that concentrations recovered from both muscle and blood reached values over the MRL in the first day within the withdrawal period. Therefore, the possibility of testing one animal containing amoxicillin concentrations higher than the MRL is rather remote. Penicillins should thus not be one of the limiting antibiotic compounds to worry about when developing new antimicrobial detection tests.

## 3. Materials and Methods

### 3.1. Chemicals and Reagents

#### 3.1.1. Antimicrobials

The antimicrobials selected for animal treatment were oxytetracycline (a tetracycline), sulfamethoxypyridazine (a sulfonamide), enrofloxacin (a quinolone), and amoxicillin (a penicillin-type antibiotic). Table S3 summarizes the source and main characteristics of each antimicrobial.

#### 3.1.2. HPLC Reagents and Standards

HPLC fluorescence grade solvents and LC–MS grade solvents were purchased from Fisher Chemical (Fisher Scientific, Leics, United Kingdom). Formic acid (98–100%) was purchased from Fisher Chemical (Fisher Scientific, Geel, Belgium). Purified water was obtained through a Milli-Q system (Millipore, Merck KGaA, Darmstadt, Germany). Amoxicillin, ciprofloxacin, enrofloxacin, oxytetracycline, and sulfamethoxypyridazine, as well as internal-standard (IS) piperacillin and demeclocicline, were purchased from VETRANAL (Sigma-Aldrich AG, Buchs, Switzerland). Enrofloxacin-d5 and sulfamethoxypyridazine-d3 were purchased from Witega (Witega, Berlin, Germany), and 4-epioxytetracycline from ACROS (Acros, Geel, Belgium). For the preparation of 0.1 M ethylenediaminetetraacetic acid (EDTA), 3.72 g of EDTA Na_2_·H_2_O (>98%, Sigma-Aldrich Chemie, Steinheim, Germany) were dissolved and made up to 100 mL with distilled water. Mobile phase for high performance liquid chromatography with fluorescence detection (HPLC-FLD) determination was phosphoric acid 25 mM (pH 3.0) and was prepared as follows: 1.7 mL of phosphoric acid 85% (for analysis, Panreac, Castellar del Vallés, Spain) was dissolved in 900 mL of purified water, pH was adjusted to 3.0 with trimethylamine (≥99%, Sigma-Aldrich Chemie), and was brought to 1 L in a volumetric flask.

Stock solutions (1 mg/mL) for each standard were prepared in appropriate solvent and kept at −20 °C: methanol for sulfamides, tetracyclines and quinolones, and water for penicillins. HPLC-FLD spiking solution contained 2 µg/mL of enrofloxacin and ciprofloxacin in methanol, and standards for external calibration were prepared in phosphoric acid 25mM (pH 3.0) at concentrations of 0.025, 0.05, 0.1, 0.25, and 0.5 µg/mL. LC–MS/MS spiking solution contained each of the studied analytes at 0.6 µg/mL, and IS spiking solution contained 1.5 µg/mL of each IS. Both solutions were prepared in methanol and maintained at −20 °C.

### 3.2. Experimental Sample Bank with Antimicrobials Administered In Vivo

To obtain samples containing antimicrobial compounds administered in vivo, 93 piglets (62 female, 31 male) with an average weight of 43.00 ± 12.79 kg were provided at treatment onset. These piglets were untreated animals that remained on the premises of the Faculty of Veterinary Sciences at the University of Zaragoza (Zaragoza, Spain) 40 days before administration of the compounds. Both during the acclimatization and withdrawal periods, animals were fed ad libitum with a special mixed feed that was free of antibiotics (ARS Alendi, S.A., Huesca, Spain), and water was provided from a separated, controlled water circuit. Piglets were raised in separate pens depending on the antibiotic administrated. 

Table S3 summarizes the main characteristics of the treatments carried out with each antimicrobial. After administration, animals were slaughtered at preset intervals within the withdrawal period in order to obtain samples containing different antimicrobial concentrations above and below the MRLs for muscle. Figure S1 indicates the slaughter days and the withdrawal periods set by the manufacturer for each antimicrobial compound administered. Between one and three piglets were slaughtered on the same day post-administration. The day of slaughter was calculated from the first day after the last antimicrobial treatment dose. Of the 93 piglets, 20 were administered with oxytetracycline, 20 with sulfamethoxypyridazine, 22 with enrofloxacin, and 16 with amoxicillin. Apart from samples containing antimicrobial compounds administered in vivo, and as a part of the sample bank, 15 of the 93 piglets were slaughtered after the acclimatization period and before treatment onset. Animals were stunned and slaughtered following the guidelines established by European Council Regulation (EC) No. 1099/2009 [39] regarding the protection of animals at the time of slaughter or killing. A penetrative captive bolt gun was used to stun the pigs, positioning it firmly against the skin, in the middle of the forehead, 2 cm above the line of the eyes, aiming towards the tail. This was followed by complete exsanguination carried out by incision in the jugular furrow at the base of the neck to sever all the major blood vessels arising from the heart. The samples obtained from them were used as blank samples for analysis.

Paired muscle and blood samples experimentally injected in vivo with oxytetracycline, sulfamethoxypyridazine, enrfloxacin, and amoxicillin, as well as untreated samples, were collected. Treated and non-treated samples were immediately prepared, as described in the following section.

#### 3.2.1. Muscle Tissue

Muscle from loins and sirloins, forequarters, and hindquarters was removed from the carcasses, separated from adiposity and fascia, split into 50 g samples, and vacuum packed. Samples were immediately frozen at −20 °C until analysis.

#### 3.2.2. Blood

Blood was collected not only from slaughtered animals, but also from living animals on each day of slaughter. In order to obtain serum, blood was coagulated at room temperature for 1 h and, after coagulum removal, was immediately centrifuged at 4 °C and 3000 rcf for 10 min on a Heraeus Megafuge 1.0R centrifuge (Heraeus, Hanau, Germany). Blood serum was aliquoted and stored in 10 mL flasks at −20 °C until analysis.

### 3.3. Ethical Approval

The experimental protocol (PI58/17) was approved by the Advisory Ethic Commission for Animal Experimentation of Zaragoza University (Zaragoza, Spain).

### 3.4. Sample Extraction

#### 3.4.1. Muscle Tissue

Approximately 50 g of muscle tissue were homogenized using a laboratory mill.

For HPLC-FLD analyses, matrix solid phase dispersion (MSPD) was used. A total of 1.00 ± 0.01 g of tissue was weighed. Internal quality control samples were spiked at this point. The sample was homogenized in a mortar with 2.5 ± 0.1 g of previously acconditioned C18. It was introduced into a 25 mL reservoir and compacted. Clean-up was made with 8 mL of hexane, and quinolones were eluted with 15 mL acetonitrile/methanol (1:1) acidified with acetic acid 1%. Eluate was evaporated to dryness and redissolved in 1 mL of mobile phase and filtered through 0.2 µm into HPLC vials prior to analysis.

The extraction procedure for LC–MS/MS determination was based on Chico et al. [40]. A total of 3.00 ± 0.05 g of homogenized muscle tissue was weighed into a 50 mL polypropylene conical test tube. All samples were spiked with IS at this point, and calibrants and internal quality control samples were prepared by spiking the corresponding amount of standard working solution. A total of 200 µL of 0.1 M EDTA was added, followed by 10 mL of 70% methanol, and samples were vortexed for 30 s, swung for 15 min, and finally centrifuged at 4500 rcf for 10 min at a temperature below 10 °C (Heraeus Multifuge X3 FR, Heraeus, Hanau, Germany). Then, 500 µL of supernatant was collected and diluted by adding 1500 µL of water. Extracts were filtered through 0.2 µm pore filters directly into LC–MS/MS vials.

#### 3.4.2. Blood

Serum was obtained from blood as previously described.

The same procedure as that followed for muscle tissue was performed, but adjusting the sample size to 1.50 ± 0.03 g and proportionally reducing reagent volumes by half. Instead of 70% methanol, we employed 70% acetonitrile, as it proved to have an improved serum protein denaturalization capability without compromising recovery. 

### 3.5. HPLC-FLD and LC–MS/MS Analyses

Quinolones in muscle tissue samples were analyzed by HPLC-FLD according to an accredited method (International Organization for Standardization (ISO) 17025:2017) [41], using an Agilent 1200 series with a fluorescence detector operating at λ_ex_ 278nm and λ_em_ 445 nm and equipped with a Zorbax Stablebond SB-C8 (5 µm, 4.6 × 250 mm) column. Mobile phase consisted of eluent A: phosphoric acid with trimethylamine buffer (pH = 3), and eluent B: acetonitrile, in isocratic mode (21:79) with a flow rate of 1 mL/min. Injected volume was 50 µL.

For quinolones in blood serum samples, a LC–MS/MS method was set up using a SCIEX Exion LC coupled to TripleQuad 6500+ triple quadrupole detector equipped with an Acquity Ultra Performance Liquid Chromatography Ethylene Bridged Hybrid (UPLC BEH) C18 (1.7 µm, 2.1 × 100 mm) column. Mobile phase consisted of eluent A: 0.1% formic acid in water, and eluent B: 0.1% formic acid in acetonitrile at a flow rate of 0.4 mL/min. Gradient started at 5%B, increased at a constant rate until 40%B in 3.75 min, then a second ramp until 95%B in 4.37 min, held constant until 5.00 min and then back to the initial 5% and held constant until 7.00 min. Injected volume was 5 µL. Analytes were detected using positive electrospray ionization (ESI+) in multiple reaction monitoring (MRM) mode, and two transitions were monitored for each compound of interest (Table S4).

A similar LC–MS/MS method was employed for the non-quinolone antimicrobials (amoxicillin, sulfamethoxypyridazine, and oxytetracycline) in both muscle and blood serum samples, using a Waters Acquity Liquid Chromatograph with TQD detector equipped with a UPLC BEH C18 column (1.7 µm, 2.1 × 100 mm). The same mobile phase and gradient conditions as in SCIEX instruments were applied. Injected volume was 10 µL. Two ESI+ MRM transitions were monitored for each compound (Table S5).

The detection limit (DL) and the limit of quantification (LOQ) of all determination techniques was 10 µg/kg, regardless of the antimicrobial compound and matrix.

### 3.6. Pharmacokinetic Parameters and Statistical Analysis

Results were obtained from at least three replicates of HPLC or LC–MS/MS analysis carried out over 2-6 muscle and blood samples obtained from the same animal and from up to three animals subjected to the same treatment and withdrawal period. Results were represented as the mean ± standard deviation using the PRISM program (GraphPad Software, Inc., San Diego, USA).

The rates of elimination (*λ_z_*) of antibiotics from muscle and blood were determined by regression analysis. The corresponding half-lives of elimination were calculated according to the following equation:

Half-life of elimination.

(1) T1/2=Ln(2)λz

where $T_{1/2}$ is the half-life of elimination and *λ_z_* is the rate of elimination.

Data were analyzed and submitted to comparison of averages via ANOVA, followed by a post-hoc Tukey test and *t*-tests with GraphPad PRISM. Differences were considered significant if *P* < 0.05.

## 4. Conclusions

Screening methods are widely used as a first step in the detection of antimicrobial substances in meat, and their adaptation to in vivo application requires obtaining samples from living animals; the easier it is to collect such samples, the more worthwhile the process. Thus, a large biological sample bank was created using samples obtained from animals treated with some of the main antimicrobial compounds used in veterinary medicine (oxytetracycline, sulfamethoxypyridazine, enrofloxacin, and amoxicillin). This bank was composed of muscle samples obtained with their paired blood samples. The correlation described between the antimicrobial concentrations in both matrixes led to improved knowledge regarding the suitability of blood as an indicator of the presence of the target antimicrobial compounds in muscle. As a result, this paper proves that blood might be a convenient matrix for the development of novel in vivo biological tests for the detection of antimicrobial compounds. Such new tools might contribute to the protection of human health while reducing economical losses and environmental contamination associated with the elimination of contaminated carcasses.

## Figures and Tables

**Table 1:** Half-lives of elimination (*T_1/2_*) calculated (Equation (1)) for oxytetracycline, sulfamethoxypyridazine, and enrofloxacin, expressed in days.

	MUSCLE				BLOOD			
	Slope	*R^2^*	$\lambda_{z}$	*T_1/2_*	Slope	*R^2^*	$\lambda_{z}$	*T_1/2_*
**Oxytetracycline**	−0.08784 ± 0.005403 ^a^	0.85	4.94	3.43	−0.08879 ± 0.007069 ^a^	0.85	5.18	3.59
**Sulfamethoxypyridazine**	−0.5494 ± 0.02370 ^a^	0.95	0.79	0.55	−0.5496 ± 0.02589 ^a^	0.92	0.79	0.55
**Enrofloxacin**	−0.3289 ± 0.01929 ^a^	0.87	1.32	0.92	−0.2213 ± 0.01639 ^b^	0.80	2.73	1.90

^a,b^ Values with different letters in superscript (a and b) within the same row are significantly different (*P* < 0.05). *R^2^:* Coefficient of determination. $\lambda_{z}$: Rate of elimination. *T_1/2_*: Half-life of elimination.

## Supplementary Materials

The following are available online https://www.mdpi.com/2079-6382/9/4/175/s1. Table S1: Antimicrobial compound concentration found in the three muscular groups tested (expressed in µg/kg). Table S2: Minimum and maximum concentration values (µg/kg) obtained in muscle and blood containing the four active compounds tested. Table S3: Source and main characteristics of the antimicrobial compounds used for the treatment of the sample bank piglets. Table S4: Monitored ions in the SCIEX TripleQuad 6500+ instrument. Table S5: Monitored ions in the Waters TQD instrument. Figure S1. Slaughter days and withdrawal periods (WP) set by the manufacturer for each antimicrobial compound administered.
