# Supplementary material for: Is Blood a Good Indicator for Detecting Antimicrobials in Meat? Evidence for the Development of In Vivo Surveillance Methods
Source: Antibiotics (Basel). 2020 Apr 12;9(4):175. doi: 10.3390/antibiotics9040175 (PMC7235904; doi:10.3390/antibiotics9040175)
Supplement: Supplementary file 1 [file antibiotics-09-00175-s001.pdf]

Table 1. Antimicrobial compound concentration found in the three muscular groups tested (expressed in μg/kg). Each value is presented as the median with the standard deviation of three chromatographic analyses of different portions of muscle from the same animal.

Active Compound	Withdrawal Period Day	Loins	Forequarters	Hindquarters
**Oxytetracycline**	Day 4	905.00±117.91[a]	971.33±53.27[a]	1005.33±55.47[a]
	Day 7	383.33±12.90[a]	410.67±27.54[a]	383.00±45.92[a]
	Day 15	125.00±15.72[a]	99.67±6.51[a]	100.00±16.00[a]
**Sulfamethoxypyridazine**	Day 2	349.00±16.46[a]	310.67±22.59[b]	351.33±20.11[a]
	Day 3	49.50±0.71[a]	43.00±0.00[a]	40.50±0.71[a]
**Enrofloxacin**	Day 2	383.00±2.00[a,b]	374.33±4.73[a]	387.33±8.50[b]
	Day 3	1060.50±2.12[a]	990.50±3.54[b]	1075.00±1.41[c]
**Amoxicillin**	Day 2	149.00±8.54[a]	20.67±1.15[b]	25.33±6.43[b]

[a,b,c] Values with different letters in superscript (a, b and c) within the same row are significantly different (*P*<0.05).

Table 2. Minimum and maximum concentration values (μg/kg) obtained in muscle and blood containing the four active compounds tested. Each value is presented as the median with the standard deviation of at least three piglets subjected to the same treatment.

		Oxytetracycline	Sulfamethoxypyridazine	Enrofloxacin	Amoxicillin
**Muscle**	**Minimum**	31.33 ± 3.21	14.00 ± 1.41	18.00 ± 2.65	13.33 ± 2.52
	**Maximum**	1005.33 ± 55.47	1179.67 ± 41.88	1858.00 ± 156.37	307.00 ± 10.39
**Blood**	**Minimum**	24.00 ± 1.73	23.67 ± 20.23	21.67 ± 1.15	19.00 ± 1.00
	**Maximum**	615.00 ± 204.47	4098.00 ± 201.34	448.50 ± 2.12	73.00 ± 6.24

Table 3. Source and main characteristics of the antimicrobial compounds used for the treatment of the sample bank piglets.

Group	Active Compound	Commercial Name	Trading House	Administration Pattern	Way of Administration	Suppression Period (days)	MRLs (µg/kg)
**B-LACTAM**	AMOXICILLIN	AMOXOIL RETARD	SYVA Lab	2 doses of 15 mg/kg separated 48 hours	Deep intramuscular	25	50
**QUINOLONE**	ENROFLOXACIN	BAYTRILUNO 100mg/ml	BAYER	2 doses of 7.5 mg/kg separated 48 hours	Deep intramuscular	12	100
**SULFAMONAMIDE**	SULFAMETHOXY-PYRIDAZINE	SULFAMETOX	S. P. VETERINARIA	-Loading dose of 40 mg/kg -Maintenance dose 20 mg/kg for 5 days	Deep intramuscular	28	100
**TETRACYCLINE**	OXITETRACYCLINE	ALAMYCIN L.A 300	KARIZOO LAB	Single dose of 30 mg/kg	Deep intramuscular	28	100

Table 4. Monitored ions in the SCIEX TripleQuad 6500+ instrument.

Compound	Precursor	Product	DP* (V)	CE* (V)
Enrofloxacin	360	342	72	30
		266	72	50
Ciprofloxacine	332	314	61	30
		231	61	50
Ciprofloxacine-d8 (IS)	340	322	61	30
Sulfamethoxypyridazine	281	156	60	25
		108	60	35
Sulfamethoxypyridazine-d3 (IS)	284	156	60	25
Oxytetracycline	461	426	65	30
		443	65	17
Demeclocycline (IS)	465	154	65	40
Amoxicillin	366	349	50	13
		114		33
Piperacilline (IS)	518	143	40	27

*DP: Declustering Potential. *CE: Collision Energy

Table 5. Monitored ions in the Waters TQD instrument.

Compound	Precursor	Product	CV* (V)	CE* (V)
Sulfamethoxypyridazine	281	92	34	30
		156	34	18
Sulfamethoxypyridazine-d3 (IS)	284	156	34	18
Oxytetracycline	461	426	30	22
		337	30	30
Demeclocycline (IS)	465	154	34	32
Amoxicillin	366	114	22	22
		208	22	12
Piperacilline (IS)	518	143	24	18

*CV: Cyclic Voltammetry. *CE: Collision Energy.

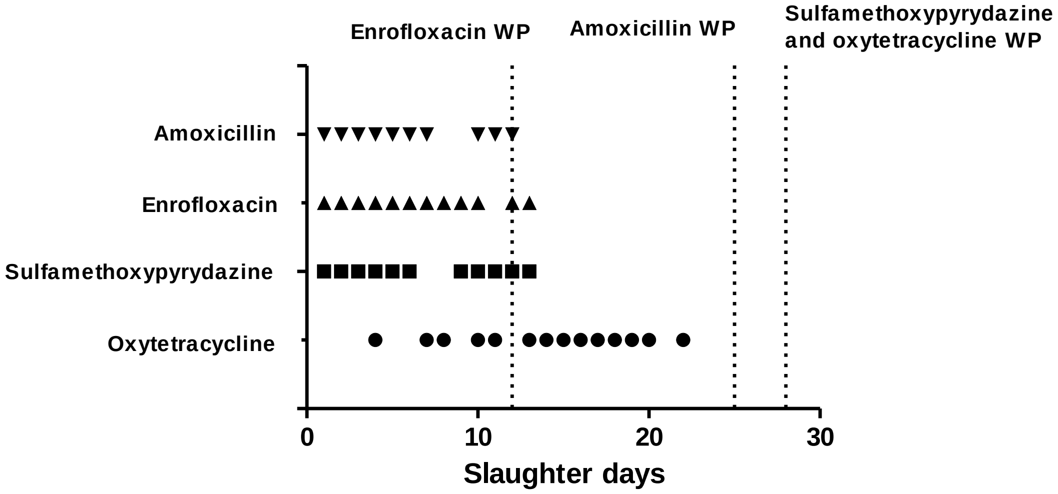

Figure 1. Slaughter days and withdrawal periods (WP) set by the manufacturer for each antimicrobial compound administered.
